# Fault Detection in Wireless Sensor Networks through the Random Forest Classifier

**DOI:** 10.3390/s19071568

**Published:** 2019-04-01

**Authors:** Zainib Noshad, Nadeem Javaid, Tanzila Saba, Zahid Wadud, Muhammad Qaiser Saleem, Mohammad Eid Alzahrani, Osama E. Sheta

**Affiliations:** 1 Department of Computer Science, COMSATS University Islamabad, Islamabad 44000, Pakistan; zainabnoshad@yahoo.com; 2 College of Computer and Information Sciences, Prince Sultan University, Riyadh 11586, Saudi Arabia; tsaba@psu.edu.sa; 3 Department of Computer Systems Engineering, University of Engineering and Technology Peshawar, Peshawar 25000, Pakistan; zahidmufti@nwfpuet.edu.pk; 4 College of Computer Science and Information Technology, Al Baha University, Al Baha 11074, Saudi Arabia; muhammad.qaiser.saleem@gmail.com (M.Q.S.); meid@bu.edu.sa (M.E.A.); 5 College of Science, Zagazig University, Zagazig 44511, Egypt; uelshata@zu.edu.eg

**Keywords:** WSNs, fault detection, machine learning, random forest, support vector machine, convolutional neural network

## Abstract

Wireless Sensor Networks (WSNs) are vulnerable to faults because of their deployment in unpredictable and hazardous environments. This makes WSN prone to failures such as software, hardware, and communication failures. Due to the sensor’s limited resources and diverse deployment fields, fault detection in WSNs has become a daunting task. To solve this problem, Support Vector Machine (SVM), Convolutional Neural Network (CNN), Stochastic Gradient Descent (SGD), Multilayer Perceptron (MLP), Random Forest (RF), and Probabilistic Neural Network (PNN) classifiers are used for classification of gain, offset, spike, data loss, out of bounds, and stuck-at faults at the sensor level. Out of six faults, two of them are induced in the datasets, i.e., spike and data loss faults. The results are compared on the basis of their Detection Accuracy (DA), True Positive Rate (TPR), Matthews Correlation Coefficients (MCC), and F1-score. In this paper, a comparative analysis is performed among the classifiers mentioned previously on real-world datasets. Simulations show that the RF algorithm secures a better fault detection rate than the rest of the classifiers.

## 1. Introduction

Wireless Sensor Networks (WSNs) are a set of independent sensor devices, which are connected by wireless channels. These are comprehending structures that deal with the surroundings closely. They are designed to perform very limited tasks. Sensors are physical devices that perform the job of assembling details about a physical object or process, including the occurrence of a change in an event like a drop in temperature or pressure [1]. WSNs have significant potential for real-time monitoring, and they are already utilized in the fields of military applications, health monitoring, industrial applications, environment surveillance, etc. Some of the constraints of WSNs include limited node energy and storage space.

WSNs are normally deployed in risky, unmonitored, and inaccessible environments, which make them more vulnerable to failures. These failures can be further divided into three categories:

- software failures,
- hardware failures, and
- communication failures.

The faults can be categorized into the following categories [2] according to the sensed data:

- Offset fault: Due to a calibration error in a sensing unit, a displacement value is added to the actual sensed data.
- Gain fault: When the change rate of sensed data is different from the expected rate.
- Stuck-at fault: When the variation in sensed data series is zero.
- Out of bounds: When the observed values are out of bounds of expected series.
- Spike fault: When the rate of change of measured time series with the predicted time series is more than the expected changing trend.
- Noise fault: When a randomly-distributed number is added to the expected value.
- Data loss fault: When there are some missing data during a specific time interval in the sensed values.
- Random fault: This is an instant error, where data are perturbed for an observation.

The acronyms are listed below in Table 1.

There are many other faults that can occur continuously and simultaneously over a period of time in sensed data, such as: calibration error, data aggregation fault, etc.

Fault detection mechanisms are considered to be of great importance to guarantee the normal functioning of WSNs. They should be precise and rapid to limit loss and to determine the status of data explicitly. However, it is a challenge to detect faults due to sensor’s constrained characteristics [1].

Many researchers have worked on the anomaly detection mechanism with different perspectives. Some techniques are distributed, centralized, or hybrid [3]. They are mostly based on dynamics, self detection, and machine learning. Machine learning is an application of artificial intelligence that provides a system with the ability to learn and improve from the experience automatically. Classification is one of the renowned approaches of data mining, which is a subset of machine learning. It separates data into different classes explicitly and helps in decision making [4]. According to the information on data, there are three categories of machine learning techniques:

- Supervised learning: Data mining techniques are applied to data with a predetermined label of the classes.
- Unsupervised learning: The techniques are applied to non-labeled data. Data are classified with no previous knowledge.
- Semi-supervised learning: This is a hybrid of supervised and unsupervised learning.

**Figure 1:**
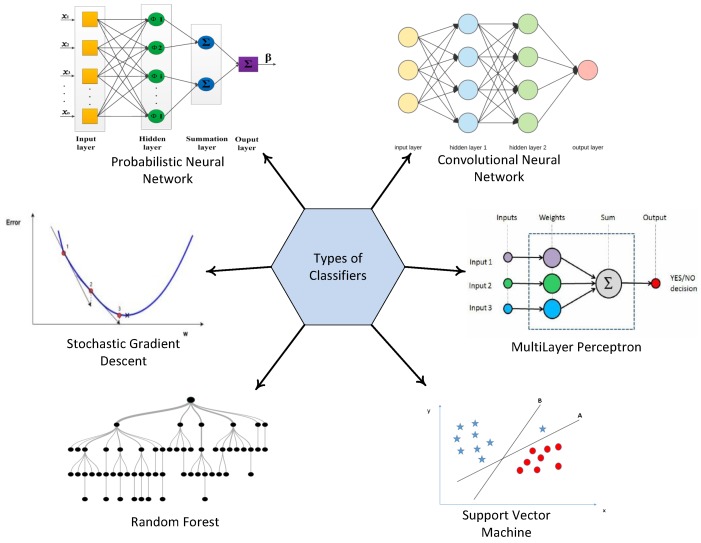
Types of classifiers.

For the purpose of fault detection, Support Vector Machine (SVM), Convolutional Neural Network (CNN), Multilayer Perceptron (MLP), Stochastic Gradient Descent (SGD), Random Forest (RF), and Probabilistic Neural Network (PNN) classifiers, as shown in Figure 1, are used to classify the sensed data into two cases, i.e., normal or abnormal case.

### 1.1. Motivation

In [1], the authors used SVM, Cloud, Spatially-Organized Distributed Echo State Network (SODESN), Bayes, and Hidden Markov Models (HMM) for classification of faulty data. Whereas, in [5], fault detection algorithms were used to detect fault in nodes and replace them in time. This ensures that a failed node does not affect and manipulate the function of the entire sensor network and system decisions. The faulty data detected can be used as a reference for the system update, which enhances the security and reliability of the network. Six classifiers are applied in this paper for the fault detection process, and results are compared on the basis of six faults; two of them are induced artificially in the datasets.

### 1.2. Challenges and Problem Statement

The fault detection process in WSNs faces many challenges due to the following reasons:

- There are very limited means and resources at the node level, which compel nodes to use classifiers [1] because they do not require complex computation.
- The sensor nodes are stationed in dangerous and risky environments, e.g., indoors, war zones, tropical storms, earthquakes, etc.
- The fault detection process [5] should be precise and rapid to avoid any loss, e.g., the process should recognize the difference between abnormal and normal cases, so that it can contain loss in the case of acquiring erroneous data that could lead to misleading results.

### 1.3. Contributions

The datasets were obtained from [1], which already had four induced faults, i.e., gain, offset, stuck-at, and out of bounds. These faults are injected at different fault rates (10%, 20%, 30%, 40%, and 50%). Each dataset consists of observation vectors Vt, and each observation vector is composed of two humidity and two temperature measurements at three successive instances. The main contributions are summarized as follows:

- In this paper, two more faults are induced in the datasets, which are listed below:
  - spike fault, and
  - data loss fault.
- Following that, six classifiers are applied on the datasets, and an extensive simulation study is conducted in our scenario to detect faults in WSN. The classifiers used are: SVM, RF, SGD, MLP, CNN, and PNN.
- The performance of classifiers is evaluated by four widely-used measures, which are stated below:
  - Detection Accuracy (DA),
  - True Positive Rate (TPR),
  - Matthews Correlation Coefficient (MCC), and
  - F1-score.

The rest of the paper is organized as follows: in Section 2, we discuss the related work on fault detection and anomaly diagnosis. Section 3 is based on an overview of faults in WSNs. Section 4 gives the basic explanation of the classifiers applied in our scenario, while Section 5 is a detailed profile of the system model. The results are included in Section 6, and the paper is concluded in Section 7.

## 2. Related Work

In the context of WSNs, there are many issues that complicate the task of fault detection at the node level. Some of these issues include; energy and resource limitations, environmental constraints, and non-stationary data. A sensor should be able to differentiate between a gain fault and a fire. This diagnosis should be efficient to restrict the loss. To confront this problem, the classification method was used. In [1], the authors have proposed a technique of classification through SVM. It divides the data into two classes: one for normal measurement and −1 for faulty measurement. The decision function is then deployed in the cluster head to classify the data.

Likewise, in [6], the authors proposed a mechanism for fault detection based on Support Vector Regression (SVR) and neighbor coordination. They tackled a similar issue of low node densities and high failure ratios. SVR is used to develop a prediction model, whereas node status is identified through mutual testing among reliable neighboring nodes.

An analysis of fault detection strategies in WSN was done in [2]. The authors divided the faults into two categories: (1) persistent faults and (2) transient faults. Persistent faults are permanent faults, whereas transient faults are temporary faults. Then, to consider the data-centric perspective, they also categorized soft faults into various categories: offset, gain, stuck-at, out of bounds, spike, and data loss faults.

In [3], an online distributed method was proposed to handle the streaming data in WSNs. The approach was based on One-Class Support Vector Machine (OCSVM) to detect anomalies over networks and to get a decentralized cost function. Instead of kernel functions, they used a random approximate function. For approximate dimensions, a sparse constraint is also added in the decentralized cost function. After that, SGD is used to minimize the cost functions and derive two distributed sets of rules. This algorithm contributed to achieving high true positive and low misdetection rates.

To determine the distance between the sensor and anchor node in WSNs, the authors in [4] proposed two algorithms. The first method estimated the distance according to the Log-Normal Shadowing Model (LNSM), while the second method used is based on Particle Swarm Optimization-Artificial Neural Network (PSO-ANN) to increase the distance estimation accuracy of the mobile node.

In [5], the authors worked on detection of link failure in WSN. The challenge is to prevent link node failures, which might end up in a disjoint set of nodes in the network. This problem leads to rapid reduction in communication. To tackle this issue, they used a FeedForward Neural Network (FFNN) with comprehensive theoretical analysis. The FFNN model learns the possible distribution of the quality of the links, and it is able to detect it once supplied to the series of Packet Drop Ratio (PDR) values.

Similarly, the authors compared SVM, naive Bayes, and Gradient Lifting Decision Tree (GBDT) to identify and classify faults in [7]. Their main focus was on noise fault, short-term fault, and fixed fault, which are caused by low battery and calibration. Whereas, in [8], machine learning techniques were discussed in the context of dynamic behavior of data. In [9], a technique was proposed for object detection using CNN. The authors highlighted the importance of machine learning in WSN applications. WSNs are prone to faults and failures because of the multiple reasons discussed above.

Data mining techniques like RF and Extreme Gradient Boost (XGBoost) are used to diagnose faults and failures in wind turbines [10]. Since the operational cost depend on component failures, it is essential to discover errors as soon as possible. RF is used to determine feature importance in the framework proposed, while XGBoost trains the classifier for each and every fault. The proposed data-driven architecture gives better results as compared to SVM, considering multidimensional data. In [11], the authors proposed a method involving two steps. The first step includes Trend Analysis incorporated with least Squares Support Vector machine (TA-SSVM). This algorithm is used for discovering faults in the sensors. The second step involves an improvised Error-Correcting Output Coding-Support Vector Machine (ECOC-SVM). It is used for identifying faults to separate failure nodes of a sensor distinctly.

A new pattern-based anomaly classifier was proposed in [12]. The Collective Contextual Anomaly Detection using Sliding Window (CCAD-SW) architecture distinguishes abnormal energy consumption patterns by overlapping SWs of smart buildings. To improve the potential of CCAD-SW, another framework was proposed called Ensemble Anomaly Detection (EAD). It elects classifiers on the basis of majority voting, and it is deployed by combining pattern-based and prediction-based anomaly classifiers to incorporate diversity among classifiers.

For the problem of a nonlinear stochastic system with WSNs, a distributed filtering technique was presented in [13]. To characterize the randomly-occurring packet losses, the Bernoulli distribution is used between WSNs and filter units. In addition to that, a fault reference model is used for enhancing the performance of the fault detection system. The Lyapunov functional approach is used to concentrate on the fault detection system’s performance and stability. Their simulation results show the effectiveness and applicability of the presented interval type-2 fuzzy model-based stochastic systems with WSNs.

In [14], the authors proposed a protocol for heterogeneous faults present in WSNs. The scheme is divided into three phases. The types of fault include hard permanent, soft permanent, intermittent, and transient faults. Packet drop and end-to-end delay are decreased through a time division-based multi-channel MAC protocol. Energy consumption in WSN is decreased through a load balancing clustering method. The second phase of fault detection of hard permanent faulty nodes is determined by a time-out status register mechanism. Analysis Of Variance (ANOVA), a statistical mechanism, is used to test differences between two or more means.

In [15], the authors considered five types of fault in a dataset, which was collected through an Arduino UNO micro-controller board. A total of 100 samples were prepared, and each sample contained 1000 data elements. SVM and neural network are used for classification, and all three kernel functions are used to train the SVM, which includes linear, polynomial, and radial basis kernel functions. A cross-validation method is adopted to overcome the issue of overfitting of the model. The results indicated that an increase in the data sample enhances the efficiency of the model, while an increase in the training sample up to 400 reduces the capability of SVM to classify stuck-at fault. Similarly, the authors discussed detection, identification, and quantification of the sensor faults in a network. The scenario they took on is of a Structural Health Monitoring (SHM) and control system through the sensor. Seven different fault types were investigated in [16] and also modeled: bias, gain, drifting, precision degradation, complete failure, noise, and constant with noise. Minimum Mean Squared Error (MMSE) is used for the estimation of each sensor. Furthermore, a sensor fault is identified and quantified by using multiple hypothesis tests using the Generalized Likelihood Ratio (GLR). The sensor network is modeled as a Gaussian process, and it is experimentally evaluated with an array of the accelerator group together on a wooden bridge.

A new hybrid approach of RFs classifier was proposed in [17] for detecting faults in rolling bearings. A rolling bearing is a bearing having loads between two races. It is used for reducing rotational friction and supports radial and axial loads. A fault in rolling bearings could result in decaying of the rotational machine’s operating conditions and capabilities. Wavelet packet decomposition is used for extracting parameters of fault features. The best set of wavelets is identified by the values of the signal-to-noise ratio and Mean Squared Error (MSE). The internal voting process of RF classifiers is then used for selecting the five dimensionless features as an input. This method was tested on practical bearing vibration signals by taking into account several fault classes. The results produced showed a record of high classification accuracy with increased efficiency and robustness in the model.

WSNs can be used in almost every domain and field now. In [18], the scenario was based on wind turbines and incipient fault detection in wind turbines. It was carried out through Deep Neural Network (DNN). Further, four steps were discussed involving a preprocessing method that considers the operational mechanism. This step is used to get rid of outliers in Supervisory Control And Data Acquisition (SCADA). RF is also used to determine the importance of variables associated with target variables. Then, SCADA data are used for training DNN, and the fault threshold is determined by the exponentially-weighted average control chart. The metric that is used to evaluate the model of DNN is the testing error of the wind turbine. This approach is found to be successful, and it was deployed in a direct drive wind turbine generator.

Link failure is one of the key issues in WSNs [19]. This occurs because of unidentified faults, which have random instances in a network. This also increases the number of faulty nodes and traffic overhead. To overcome such a problem, a cluster that is based on a fault-tolerant technique using the Genetic Algorithm (GA) is proposed. A number of backup nodes are selected for each cluster head through GA. It is based on sponsored coverage and residual energy parameters. The results showed that the proposed technique minimizes the energy loss and overhead by detecting faults that occur in cluster heads and members.

In [20], an approach was suggested for automatic crack detection and classification in subway tunnel safety monitoring. The process was described in three phases: The first phase consists of storing the digital images of the tunnel surface through industrial cameras. In the second phase, by utilizing morphological image processing techniques and threshold operations, the dark areas with potential crack defects are separated from the grayscale stored images. The third phase is a feature extraction process; a distance histogram is used for describing the difference between cracks and other peripheral objects.

To address the challenges of WSN and the renowned problem of the decentralized multi-class classification fusion problem, a simple, but effective decision fusion rule based on belief function theory was proposed in [21]. Based on the classifier’s training output confusion matrix and real-time observations, Basic Belief Assignments (BBAs) are constructed for each sensor. Furthermore, Dempster’s combinational rule is used to formulate global BBA in the fusion center, which has simplified decision making operations. The experimental results showed that the proposed fusion rule performs better in terms of fusion accuracy for WSNs.

In [22], the authors addressed the problem of distributed sensors’ failure detection in networks through a novel technique based on a gossip algorithm and group testing principles. Likewise, in [23], a distributed fault detection algorithm was proposed for WSNs. Faulty sensor nodes are identified based on comparisons between the neighboring node and dissemination of the decision made at each node.

The authors of [24] proposed another distributed fault detection algorithm. In the proposed technique, a node first collects the measurements of its neighborhood and processes them to decide whether they contain outliers or not, and then, the results are broadcast. Similarly, in [25], the authors proposed and analyzed the performance of two distributed algorithms to help each node in determining whether it is equipped with a defective sensor or not.

Authors in [26] have proposed depth and reliability aware delay sensitive, interference aware, and cooperative routing protocols which is to maximize the through put of the network while decreasing the end-to-end delay. In [27], authors have worked upon opportunistic and geographic routing protocol based on interference avoidance for underwater WSNs. Likewise in [28,29], authors have worked on void hole avoidance, collision, and reliable data delivery in underwater WSNs. Whereas in [30], the authors have worked on region based routing protocols in underwater WSNs. Their proposed technique is used for forward and amplify technique over Rayleigh faded channels in underwater WSNs. A brief summary of related work with its limitations is given in Table 2.

## 3. Faults in WSNs

As discussed in the previous section, the scenario in this paper includes six faults [1,8]. These faults lie in the data-centric perspective of WSNs, while calibration fault and battery failure are grouped into the system-centric perspective. Since WSNs are deployed in harsh and hostile circumstances, e.g., thunderstorm, snowstorm, rain, etc., they are likely to have frequent and unexpected errors. The occurrence of faults [2] during normal operation can result in harsh consequences involving the loss of human life, as well as economic and environmental losses. To prevent these calamitous situations, faults are categorized into multiple classes. The core reason to categorize faults is to detect them at an early stage so that appropriate measures can be taken to resolve them or to opt for an alternative solution to prevent any critical situation. A description of fault categories is outlined below according to the collected data.

The gathered data are modeled by the triplet *d(n, t, f(t))*; where *f(t)* represents the sensed value at time *t* of node *n*. This equation is represented in Equation (1):

(1) f(t)=α+βx+η

In the above equation, sensed data f(t) are modeled as α, which is offset, β represents gain, while η is the noise present in the data [1].

### 3.1. Gain Fault

As the name suggests, the rate of change of data is more than the expected reading in the gain fault [1]. This happens because of the multiplicative nature of β with data point *x*. This fault is represented in Equation (2).

(2) $$x^{\prime }=\beta x+\eta$$

where β represents the constant, which is multiplied with the normal reading.

### 3.2. Offset Fault

Offset fault is when collected data are out of line [1]. This happens when a constant is added to expected data due to the bad calibration of the sensing unit. Offset fault can be modeled as follows:

(3) $$x^{\prime }=\alpha +x+\eta$$

where α represents the constant value added in the normal reading.

### 3.3. Stuck-at Fault

A stuck-at fault is defined [1] as a series of data values that experiences zero or almost zero variation for a period of time greater than expected. The zero variation must also be counter to the expected behavior of the phenomenon. This fault is modeled in Equation (4) as:

(4) $$x^{\prime }=\alpha$$

where $x^{\prime }$ is the non-faulty data gathered by the node at time *t*.

### 3.4. Spike Fault

When the rate of change of measured time series with forecasted time series is more than the expected changing trend, spikes are created, which is categorized as spike fault. It can be modeled [3] in the way presented in Equation (5).

(5) $$\frac{|f\left(t\right)-f^{p}\left(t\right)|}{t}>\lambda$$

where f(t) is the original data, $f^{p}\left(t\right)$ is the predicted time series at time *t*, while λ represents the normal changing trend in sensed data. The spike fault was also discussed in [8].

### 3.5. Data Loss Fault

A data loss fault [2] occurs when the sensed data have missing values from the series of a particular node. This could happen because of hardware failure, calibration fault, or battery failure. Equation (6) represents the data loss fault.

(6) f(t)=Φ,t>τ

where τ is the maximum time that is required for sensing data and Φ is a null set.

### 3.6. Out of Bounds

This is when sensed data lie beyond the threshold that is set for the problem requirement [3]. The limitation of out of bounds fault is defined in Equation (7).

(7) $$x^{\prime }>\theta \;or\;x^{\prime }<\theta_{1}$$

In the equation mentioned above, there is a threshold defined for data variable $x^{\prime }$ where θ and $\theta_{1}$ represent the established range.

## 4. Classifiers

Classifiers are used to classify a new observation into a set of categories, on the basis of a training set of data containing the observations. From the literature review of multiple research papers on fault detection [1,9,10,14], we selected some classifiers to implement in our scenario. In [1], SVM was used for fault detection in WSN, and CNN was used for binary classification and object detection in [9], whereas the authors in [10] used the RF method to detect and classify faults, as well as to rank the features in wind turbines. In [14], PNN was used to classify different kinds of nodes in the network. This helped us narrow down six classifiers that are used for fault detection by binary classification. The basic explanation of these classifiers is given in the subsections below.

### 4.1. SVM

SVM [1] is a non-probabilistic classifier formally defined by a separating hyperplane. The given training data are labeled (supervised learning), and the algorithm draws an ideal hyperplane, which has the maximum distance from the support vectors. In two-dimensional space, this hyperplane is a line dividing a plane into two classes. The tuning parameters of the SVM classifier are the epsilon (ε), regularization, and kernel parameters.

### 4.2. MLP

MLP [14] is a class of FeedForward Artificial Neural Networks (FFANN). It consists of at least three layers of nodes. Except for the input nodes, each node is a neuron that uses a nonlinear activation function. MLP utilizes a supervised learning technique called back propagation for training.

### 4.3. CNN

CNN [9], like all neural networks, is inspired by the brain’s ability to detect and process text, images, and videos. It is made up of neurons with learnable weights and biases. It has an input layer, an output layer, and various hidden layers.

Each neuron receives several inputs; it takes a weighted sum over them, passes it through the activation function, and responds with an output. There are five steps of CNN, which are as follows:

- In the convolutional step, there are three important parts to mention: the input, the feature detector, and the feature map. The main objective of this step is to reduce the size of the input, which would eventually make the process faster and easier.
- The rectified linear unit is the rectifier function (activation function) that increases the non-linearity in CNN.
- Pooling enables CNN to detect features in various images, irrespective of the difference in lighting or their variant angles.
- Once the pooled feature map is obtained, the next step is to flatten it. Flattening means converting the entire pooled feature map image into a single column, which is then fed to the neural network for further processing.
- The last layer is made up of an input layer, a fully-connected layer, and an output layer. The output layer generates the predicted class. The information is passed through the network, and the error of prediction is calculated. The error is then transmitted back to the network for improvement in prediction.

### 4.4. RF

The RF method or random decision forest is a collective learning method for classification, regression, and other tasks. It operates by creating a cluster of decision trees at training time and producing a class that is the mean prediction of individual trees. In [10] RF, there is a directly proportional relationship between the number of trees in the forest and the resultant accuracy. There are two steps in the RF algorithm, which are as follows:

- The first step is to create an RF tree. It is further elaborated in the following five stages:
  - *K* number of random features are selected from total features *m*, where *K* is less than *m*,
  - within the selected features, node *d* is determined using the best split point,
  - those nodes are further distributed into daughter nodes through best split,
  - the first three steps are repeated until *l* number of nodes are obtained,
  - all of the above steps are repeated for *n* times to achieve *p* number of trees, where *n* is not equal to *p*.
- The next step is to classify the data based on an RF tree that has been created in the first step. It has the following stages:
  - with the rules created for each randomly-formulated decision tree and test features, data are classified,
  - the votes are calculated for each target value,
  - the highest voted prediction target is considered to be the final result of the RF algorithm.

### 4.5. SGD

An estimator algorithm implements regularized linear models with SGD learning [33]; the gradient of the loss is estimated each sample at a time, and the model is updated along the way with a decreasing strength schedule. SGD allows mini-batch (online/out-of-core) learning. To obtain the best results using the default learning rate schedule, the data should have zero mean and unit variance. The regularizer is a penalty added to the loss function that shrinks the model parameters toward a zero vector. This is accomplished by using either the squared Euclidean Norm (EN) L2, the Absolute Norm (AN) L1, or a combination of both, which is called elastic net. If the parameter update crosses a 0.0 value because of the regularizer, the update is truncated to 0.0. This allows the learning of sparse models and achieving online feature selection.

### 4.6. PNN

PNN [14] belongs to the family of neural networks. It is sensitive in scenarios when one input feature has a higher value than the other one. The input variables should have a similar scale, otherwise they should be normalized before use. PNN is an efficient algorithm for smaller datasets and classification problems. The Probability Distributed Function (PDF) used in PNN is calculated by the Parzen Window (PW), which is based on Kernel Density Estimation (KDE). In PNN, layers are organized in the multilayered FeedForward Network (FFN) at four levels, which are as follows:

- Input layer: The first layer is the initiating layer of PNN. It has multiple neurons where each neuron represents a predictor variable. There are *N* number of groups, and N−1 neurons are used as categorical variables. Then, the range is standardized by dividing the subtracted median by the interquartile range. These values are used as an input for the next layer of neurons, which is a pattern layer.
- Pattern layer: This is the second layer of PNN. Each case contains a single neuron in the training dataset. The target value and predictor variables are stored together in a case. Then, a private neuron calculates the Euclidean Distance (ED) from the center point of all neurons. This process is repeated for each and every case. After that, ED is used with the radial basis kernel function along with the sigma values.
- Summation layer: The output of the pattern layer is a pattern neuron that is used as an input for the summation layer. In PNN, each category of target variables has one pattern neuron. After that, every hidden neuron stores the actual target category of each training case; these weighted values are streamed in the pattern neurons that are in compliance with the hidden neuron’s category. These values are added in pattern neurons for the determined class.
- Output layer: The weighted votes are compared in the output layer. The selection criteria for determining which vote should be used for predicting the target category are based on the comparison of weighted votes for each target class that is calculated in the second layer.

## 5. System Model

**Figure 2:**
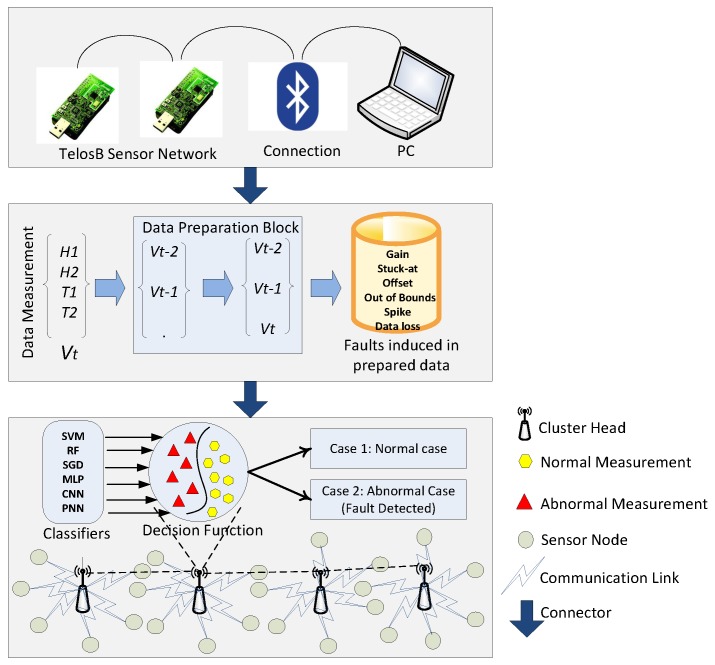
System model of fault detection.

The proposed scenario includes two TelosB mote sensors for assembling measurements. As presented in Figure 2, the system model can be divided into three phases, which are as follows:

### 5.1. Phase 1

The sensed readings are used as an input for the data preparation block that prepares a new observation vector from each new data measurement *Vt*. *Vt* is composed of two humidity measurements, i.e., *H1*, *H2*, and two temperature measurements, i.e., *T1*, *T2*. Three successive data measurements *Vt*, *Vt-1*, *Vt-2* aggregate to form a new observation vector.

### 5.2. Phase 2

The next phase is the infusion of faults in the dataset. Datasets with four faults, which are gain, stuck-at, offset, and out of bounds, were taken from [1], whereas the other two faults, i.e., spike and data loss faults, were introduced in the second phase of the system model.

### 5.3. Phase 3

WSNs are composed of multiple clusters of nodes interconnected with each other. Each cluster has a cluster head, which communicates between the layers of the network and with other nodes. This cluster head is shown with a zoomed out view in our system model. Labeled datasets are used in the learning phase.

In the third phase, the classifiers that are used for classification, i.e., SVM, CNN, SGD, MLP, RF, and PNN, are deployed in the cluster head. These techniques use observation vectors to formulate a decision function. Since, the algorithm used for deploying the decision function in the cluster head is composed of an uncomplicated implementation, the process is not computationally expensive. After deployment of the decision function, data are classified into two classes. If the result is positive, the data belong to the first class (normal case), otherwise data are considered as an abnormal case (faulty case).

## 6. Simulations and Results

To evaluate the performance of classifiers, we have performed our simulations in Python. The datasets were used as an input for the simulator. The simulator was running on the system Intel core i3, 4 GB RAM, and 500 GB of storage. The detail of the datasets and simulations are discussed in the section below.

### 6.1. Datasets

The data were gathered from two outdoor multi-hop sensors. They were the sensed data of temperature and humidity. Each vector was composed of data collected at three successive instances *t0*, *t1*, *t2*, and each instance was constructed from two temperature measurements and two humidity measurements *T1*, *T2* and *H1*, *H2*. After that, different type of faults (offset, gain, stuck-at, out of bounds, spike, and data loss) were induced randomly at different rates (10%, 20%, 30%, 40%, and 50%). A total of 40 datasets were prepared with a set of 9566 observations (vectors) and 12 dimensions each. The datasets were labeled with a target column marked one for normal and −1 for abnormal observations.

### 6.2. Results

To evaluate the performance of SVM, RF, MLP, SGD, CNN, and PNN, four metrics were used for comparison. The first metric was DA [1,7], and it is formulated as given in Equation (8):

(8) $$DA=\frac{\mathrm{Number}\;\mathrm{of}\;\mathrm{faulty}\;\mathrm{observations}\;\mathrm{detected}}{\mathrm{Total}\;\mathrm{number}\;\mathrm{of}\;\mathrm{faulty}\;\mathrm{observations}\;}$$

The other metric used was TPR [7]. It is the measure of actual positives, which are correctly identified. It is defined as follows:

(9) $$TPR=\frac{TP}{TP+FN}$$

In Equation (9), True Positive (TP) refers to the measurements that predict true positives, while False Negatives (FN) are those measurements that are incorrectly claimed as negative.

The third metric used is Matthews Correlation Coefficient (MCC) [34], which is used to rank fault diagnostic schemes according to their DA. The range of MCC is between −1 and one. The technique that scores −1 is an incompatible algorithm; zero stands for similarity to random prediction; while one stands for an ideal technique. A closer value to +1 states a very strong relationship between reality and the test. MCC is defined as follows:

(10) $$MCC=\frac{TP\times TN-FP\times FN}{\sqrt{\left(TP+FP)(TP+FN)(TN+FP)(TN+FN\right)}}$$

In Equation (10), True Negative (TN) declares correctly-defined non-faulty nodes, while False Positive (FP) is defined as the number of faulty nodes incorrectly identified as faulty nodes. MCC was applied on all six classifiers that were implemented in this scenario to test the accuracy of binary classification and rank algorithms.

The fourth metric used for statistical performance evaluation is the F1-score. The F1-score [35] is fundamentally the harmonic mean of precision and recall. It is used as a statistical measure to rate the performance of an individual classifier on the basis of FN and FP. Precision is defined as the accuracy of judgment, whereas recall is detecting all the instances in a sample that bear witness to a certain attribute, i.e., faulty or non-faulty. The F1-score is defined as below in Equation (11);

(11) $$F1-Score=\frac{2\times Precision\times Recall}{Precision+Recall}$$

Table 3 shows the ranking of all the classifiers based on the MCC score. The values between −0.10 and −0.50 indicate weak classifiers; therefore, they have a poor relation with reality. In our scenario, RF and SVM were considered compatible because of the acquired MCC values. Since RF was closest to +1 as compared to SVM, it was ranked as first, while SVM was graded as the second compatible algorithm, with a score of 0.65, among all the classifiers.

**Figure 3:**
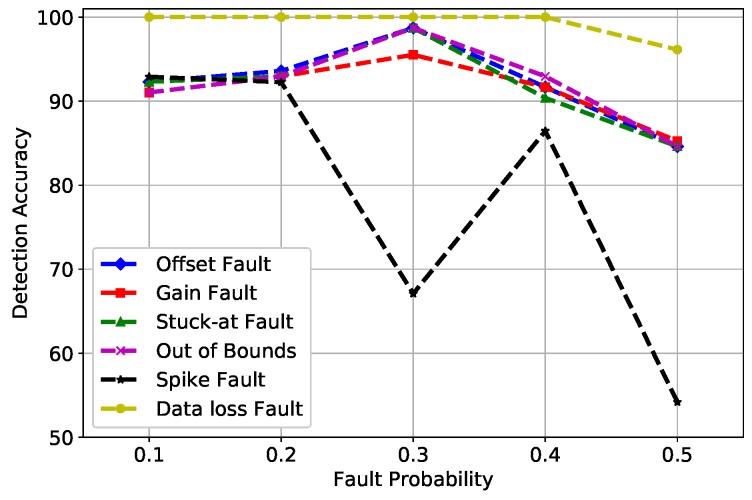
SVM detection accuracy of fault types.

Figure 3 shows the DA of SVM for all six fault types that were induced at different fault rates (10%, 20%, 30%, 40%, and 50%). The best learning rates were obtained at σ = 0.5 and *C* = 1 after an extensive parameter tuning, where the kernel function of SVM has a parameter that significantly impacts the performance of the classifier, referred to as σ, whereas *C* refers to the cost function that controls the influence of each support vector in SVM. The decrease of DA in gain and spike faults is because of the constant value β multiplied by the normal reading. The spike fault at 0.3 probability showed an usual decrease in DA because at 30%, outliers were diligent at detecting, while at 50%, the fault rate was increased and SVM did not perform well with the increased number of outliers in large datasets. Furthermore, the performance of SVM depends on the distance of support vectors from the decision surface; therefore, at a 50% fault rate, the distance decreased from the margin, which illustrates the unusual behavior of the graph.

**Figure 4:**
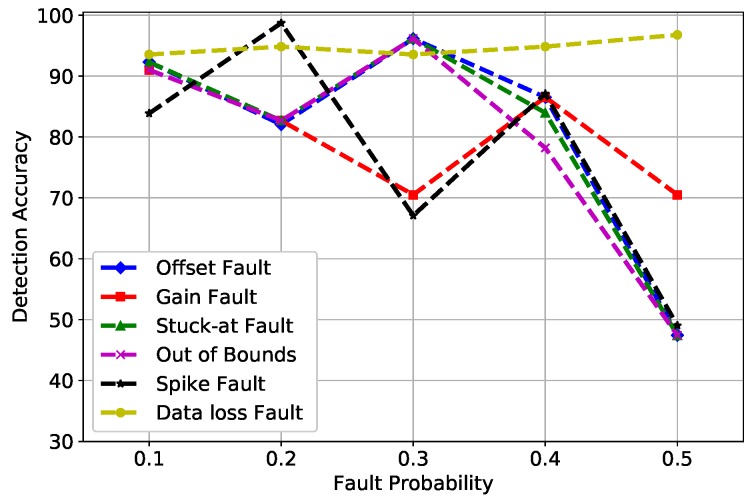
MLP detection accuracy of fault types.

In Figure 4, MLP uses a back propagation method for training, which causes network paralysis and slow convergence. In network paralysis, the weights are adjusted to a very large value while training, which forces all units to work at extreme values even when the activation function is small. Whereas, in slow convergence, the classifier requires recurrent presentation of the input pattern continuously. Both of these factors were responsible for the variance in DA at multiple fault rates. The spike fault showed some unusual oscillations because MLP works well with linearly-separable data, whereas the fault induced was at a random rate. Due to this reason, MLP behaved strange at different fault rates.

**Figure 5:**
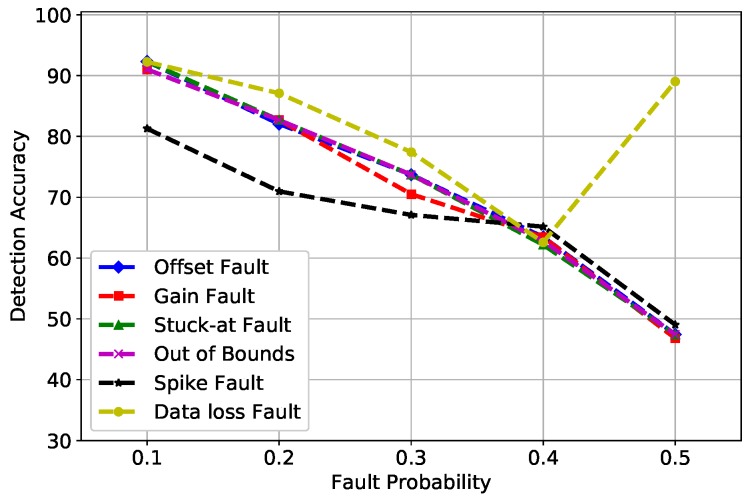
SGD detection accuracy of fault types.

Figure 5 shows that, as the fault rate increased, the DA of SGD decreased. SGD is sensitive to feature scaling; however, we have not scaled our data, and because of a continuous change induced in the sensor measurements, the accuracy of SGD dropped by 10% at every fault rate consecutively. Another problem faced by SGD in our proposed scenario is that, after weights were updated at every layer, the training set started to learn the precise sensor data to match each score. Through this, it worked really well on training data; however, in the testing phase, it failed to show the same accuracy as in the training phase.

**Figure 6:**
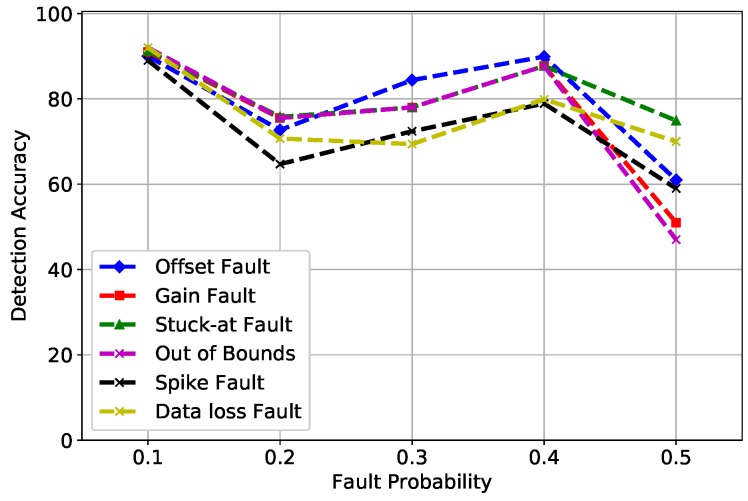
CNN detection accuracy of fault types.

Figure 6 shows the trend of CNN. At a 0.5 fault probability, the DA of stuck-at, offset, gain, out of bounds, data loss, and spike fault was 74.9%, 61.0%, 51%, 47%, 71.6%, and 60.6%, respectively. The low DA of CNN was because after pooling, the feature map was flattened, which ended up reducing the feature dimensions. As we only had 12 dimensions, CNN reduced the features to half, which caused DA to drop at a higher fault rate.

**Figure 7:**
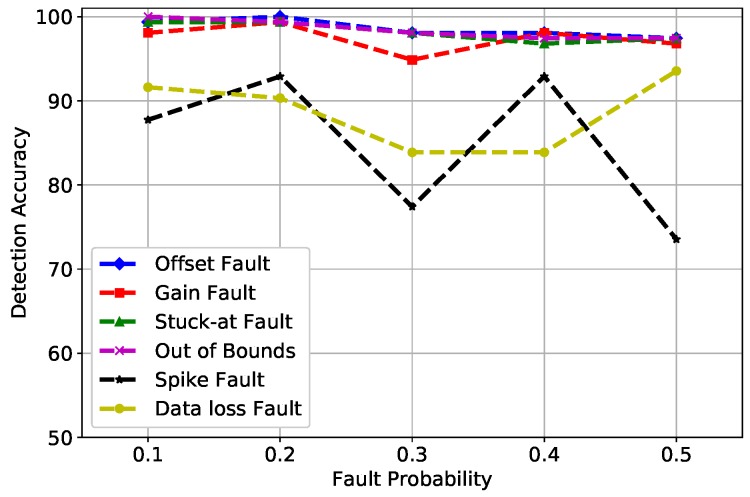
RF detection accuracy of fault types.

In Figure 7, the fault probability at 0.1 and 0.2 indicates that the DA of gain, offset, stuck-at, and out of bounds fault was between 98% and 100%. However, the spike fault was showing an unusual behavior at 0.3 and 0.5 fault rates because this is the rate of change of measured time series with forecasted time series, which is more than the expected changing trend, as explained in Equation (5); therefore, spike fault was difficult to detect with high accuracy. Furthermore, when enough trees were generated in the forest, RF did not over fit the model and generated good DA. The default value of the *n* estimator used by RF was 100; however, the best learning rates obtained in our scenario were at *n* estimators = 1000, which was the number of trees in the forest. Hence, it can be concluded that as the number of trees increased, the DA of RF improved.

**Figure 8:**
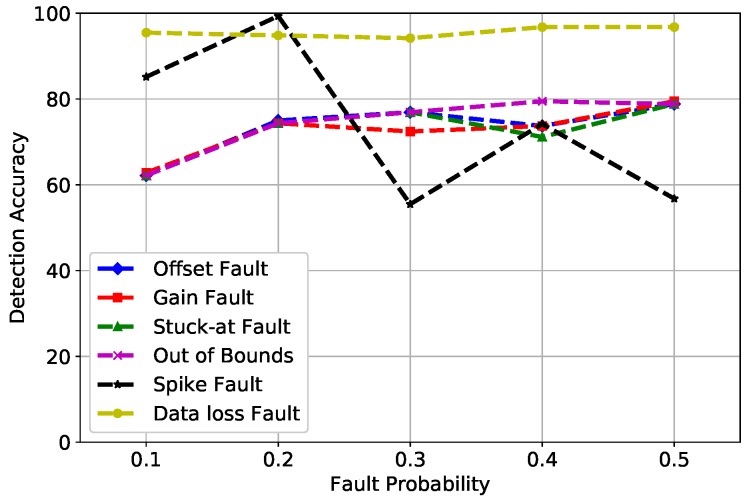
PNN detection accuracy of fault types.

PNN is widely used for classification and pattern recognition. In Figure 8, DA of PNN is mapped against fault probability. There were three parameters of PNN that were considered in our scenario: standard Deviation (STD), verbose, and batch size, which were set at 90, true, and none, respectively. The accuracy of PNN fluctuated between a range of 60 and 80%. The summation of PDFs in the third layer of PNN did not produce good results due to which faults were not classified as meticulously as compared to SVM and RF.

**Figure 9:**
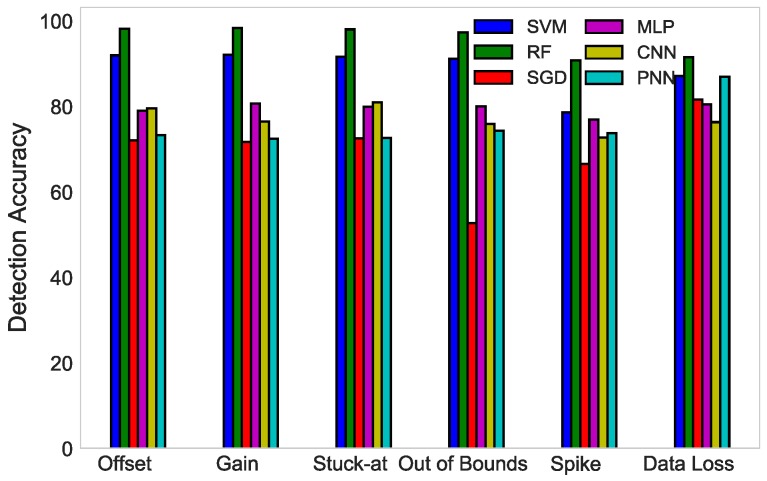
DA comparison of SVM, RF, SGD, MLP, CNN, and PNN detection of four types of fault.

Figure 9 compares the DA of SVM, RF, MLP, CNN, SGD, and PNN for six fault types. It can be deduced from the figure that SVM was superior to MLP, SGD, CNN, and PNN in the detection of all six faults; however, due to the large number of instances used in this scenario for the evaluation of the randomization concept explained in Section 4, RF outperformed the rest of the classifiers. Whereas, SVM only works well with reasonably refined and smaller datasets, this is why RF demonstrated better results than SVM.

**Figure 10:**
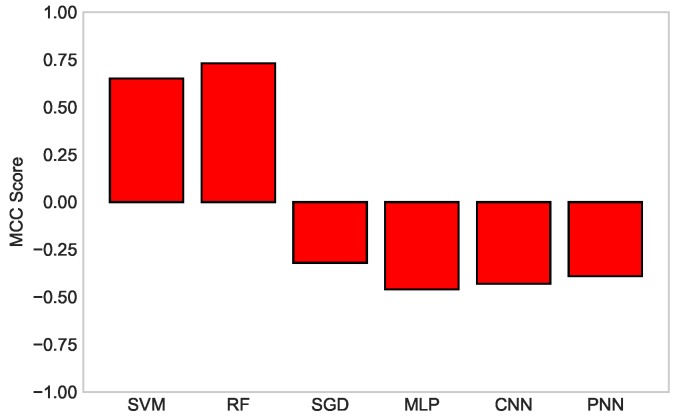
MCC score comparison of SVM, RF, SGD, MLP, CNN, and PNN classifiers.

In Figure 10, a comparison among all the classifiers is carried out on the basis of their MCC score. The bars explicitly depict the ranking of the algorithm used for classification. RF maintained its position at the first rank, whereas MLP secured the last rank among all the classifiers. The scores are listed with the ranks in Table 3 with the explanation provided below it.

**Figure 11:**
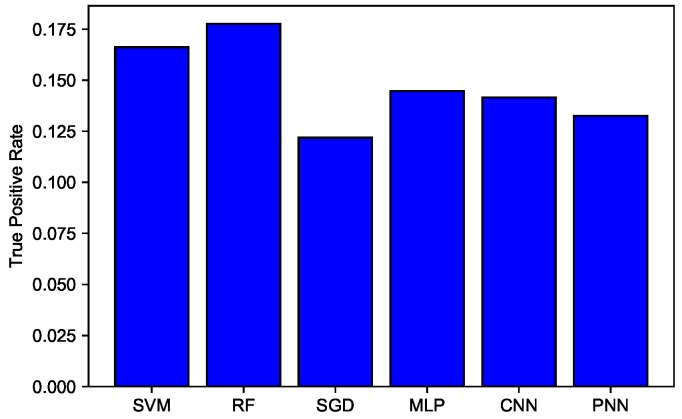
TPR of SVM, RF, SGD, MLP, CNN, and PNN.

In Figure 11, a comparison of TPR among SVM, RF, MLP, SGD, CNN, and PNN is given. SVM gave 0.166 TPR, which was higher relative to other classifiers, but clearly, RF beat all other classifiers with 0.177 TPR. This indicates that RF accurately detected more actual positives.

**Figure 12:**
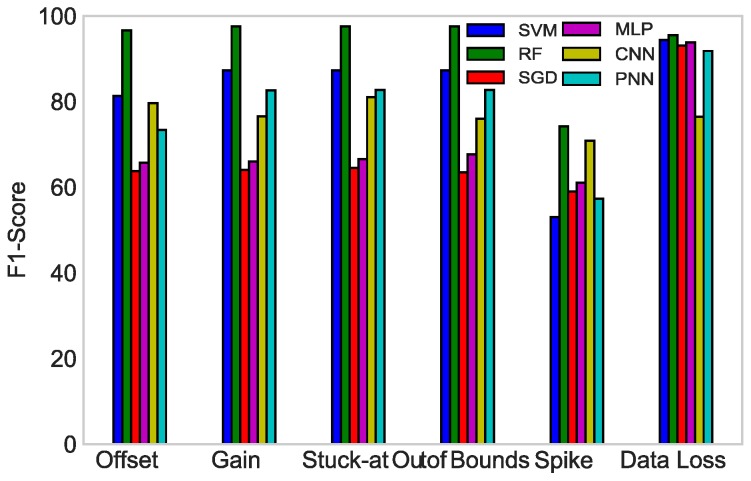
F1-score of SVM, RF, SGD, MLP, CNN, and PNN.

In Figure 12, the F1-scores of all the classifiers against each fault type are compared with each other. To create a balanced classification model with the ideal values of recall and precision, the F1-score was maximized. In the bar graph, RF had the highest value of F1-score in all the fault types, giving an average of 93.15%. Therefore, RF outperformed all the classifiers in our proposed scenario.

## 7. Conclusions and Future Work

In this paper, the research work is preceded by a dataset preparation block. The datasets consist of observation vectors Vt, which are composed of two humidity, i.e., *H1*, *H2*, and two temperature measurements, i.e., *T1*, *T2*, at three successive instances. After that, the datasets are injected with two faults, i.e., spike and data loss faults, at different fault rates (10%, 20%, 30%, 40%, and 50%). Following that, six classifiers, i.e., SVM, CNN, MLP, SGD, RF, and PNN, are applied on outdoor data collected from multi-hop WSNs. The classifiers are then evaluated on the basis of four different performance metrics: DA, TPR, MCC, and F1-score. The extensive simulation results show that RF outperformed in terms of DA and TPR. It also emerged as victorious in terms of MCC and F1-score as compared to the rest of the classifiers.

For future work, we will use the same classifiers to predict an upcoming fault in data so that a mechanism can be developed to prevent faults. Furthermore, we will work on fault identification in WSNs, which will accurately identify and then detect faults on the sensor (node) level. The robustness of the RF algorithm can also be checked by increasing the number of sensors. This will help to understand how resilient a WSN is against attacks.

## Figures and Tables

**Table 1:** List of acronyms.

AN	Absolute Norm
ANOVA	Analysis Of Variance
BBA	Basic Belief Assignment
CCAD-SW	Collective Contextual Anomaly Detection using Sliding Window
CNN	Convolutional Neural Network
CMOS	Complementary Metal Oxide Semiconductor
DNN	Deep Neural Network
DA	Detection Accuracy
ECOC-SVM	Error-Correcting Output Coding-Support Vector Machine
EN	Euclidean Norm
ED	Euclidean Distance
EAD	Ensemble Anomaly Detection
FFNN	FeedFoward Neural Network
GA	Genetic Algorithm
GLDT	Gradient Lifting Decision Tree
GLR	Generalized Likelihood Ratio
KDE	Kernel Density Estimation
LNSM	Log-Normal Shadowing Model
MMSE	Minimum Mean Squared Error
MSE	Mean Squared Error
MLP	Multilayer Perceptron
MCC	Matthews Correlation Coefficients
OCSVM	One-Class Support Vector Machine
PSO-ANN	Particle Swarm Optimization Artificial Neural Network
PDF	Probability Distributed Function
PNN	Probabilistic Neural Network
PDR	Packet Drop Ratio
RF	Random Forest
SVM	Support Vector Machine
SGD	Stochastic Gradient Descent
SVR	Support Vector Regression
SHM	Structural Health Monitoring
SCADA	Supervisory Control And Data Acquisition
TPR	True Positive Rate
TA-SSVM	Trend Analysis least Squares Support Vector Machine
WSNs	Wireless Sensor Networks
XGBoost	Extreme Gradient Boost

**Table 2:** Summary of related work.

Techniques	Contributions	Limitations	Future Work
SVM and Statistical Learning Theory [1]	Fault detection in WSNs	Upcoming faults are not identified, predicted, and quantified; it does not consider single-hop indoor datasets	Forecasting of upcoming faults
OCSVM and SGD [3]	Anomaly detection in WSNs	No proposed scheme for anomaly identification mechanism	Multi-class, one-class classification problems for efficient anomaly detection
LNSM and PSO-ANN [4]	Measured the RSSI in real environments, determined the distance between sensor and anchor nodes in WSNs, and improved the accuracy of estimated distance	For outdoor environments, the effect of anchor node density on localization accuracy was not calculated	Algorithms can be evaluated through more metrics such as MAPE and MAE
SVR and Neighbor Coordination [6]	Fault detection for WSNs	The proposed algorithm is specific to meteorological elements, and there is no comparison with any other classifier	Comparisons with more than two classifiers for validation
CNN [9]	Image classification and object detection	No mechanism for fault identification	Fault identification classifiers can be incorporated with CNN
RF and XGBoost [10]	To detect fault in wind turbines	The number of features is limited to 10	More comprehensive training data in multiple wind turbine working conditions, including all the unconsidered faults
SVM and TA [11]	Improvised sensor fault diagnosis	Single type of ECOC coding matrix in both feature extraction and fault classification	Parameter optimization processes
RF and SVR [12]	Anomaly detection in energy consumption sensors	No comparison with other hybrid classifiers that are trained on different datasets and features	More robust voting techniques should be explored such as weighted voting
Stochastic systems [13]	Distributed soft fault detection in WSNs	Limitation of the network-induced delay and event-triggered mechanism	Non-linear systems with complex and limited communication
PNN [14]	Heterogeneous fault diagnosis	No variation in the classifier	Further, this methodology can be applied on body area sensor networks, vehicular ad-hoc networks, and UWSN
SVM and Statistical Time-Domain Features [15]	Sensor fault classification	The scheme is implemented on only five faults	More faults should be considered
MMSE, Multiple Hypothesis Test, and GLR Test [16]	Wireless sensor fault detection, identification, and quantification	A large number of time-synchronized samples are required for MMSE identification	Future work in energy efficiency of WSNs
DNN and Conventional RF [17]	Fault detection in a direct drive wind turbine	No evaluation through comparisons	Comparison of DNN with other neural network algorithms
GA [18]	Fault detection in cluster head and cluster members	Limited use of the optimization technique	For better evaluation, multiple optimization techniques can be used to validate the scheme
LSVM [31]	A data-driven framework for reliable link classification of WSNs	Not suitable for unsupervised data	The technique can be enhanced for anomaly identification in link failure
SVM, GA, and Tukey Test [32]	To adjust the transmission rate in WSNs	No scheme for identifying and detecting anomalies in the transmission or congestion rate	Multi-classification to detect faulty nodes

**Table 3:** MCC of SVM, RFC, SGD, MLP, CNN, and PNN.

Technique	Matthews Correlation Coefficient (MCC)	Rank
SVM	0.65	2
RF	0.73	1
SGD	−0.32	6
MLP	−0.46	3
CNN	−0.43	4
PNN	−0.39	5
